# TPU Wrapped Nanocomposite Films with Nickel and Magnetite Nanoparticles for Effective UV and EMI Shielding

**DOI:** 10.3390/nano16150963

**Published:** 2026-08-05

**Authors:** Ogirala Venkata Pandu Ranga Sivakumar, Sundaramoorthy Arunmetha, Nattanmai Raman Dhineshbabu, Arunkumar Jayakumar, Sengottaiyan Shanmugan

**Affiliations:** 1 Department of Electronics and Communication Engineering, Koneru Lakshmaiah Education Foundation, Green Fields, Vaddeswaram 522502, Andhra Pradesh, India; 2 Department of Electronics and Communication Engineering, T. John Institute of Technology, Bengaluru 560083, Karnataka, India; dhineshbabu@tjohngroup.com; 3 St. Peter’s Institute of Higher Education and Research, Avadi, Chennai 600054, Tamil Nadu, India; arunkumarj.eee@spiher.ac.in; 4 Research Centre for Solar Energy, Department of Integrated Research & Discovery-Physics, Koneru Lakshmaiah Education Foundation, Green Fields, Vaddeswaram 522502, Andhra Pradesh, India

**Keywords:** thermoplastic polyurethane polymers, nickel nanoparticles, SWCNT, ferromagnetic materials, shielding effectiveness against UV and EMI

## Abstract

In recent years, multifunctional composite nanoparticles have garnered substantial attention across multiple fields, from medicine to environmental science and the food industry, owing to their superior physicochemical properties. The synching of Ni nanoparticles by chemical reduction with nickel chloride as the source, and Fe_3_O_4_ nanoparticles by the co-precipitation method, with Fe^2+^ and Fe^3+^ as salts, is the focus of this study. Silane was used for the surface modification of Fe_3_O_4_ nanoparticles, while sulfuric acid was used to modify the SMCNT. A composite in PVDF based on the blend of Ni and modified Fe_3_O_4_/single-walled carbon tube (SWCNT) was used as an additive. Moreover, thermoplastic polyurethane (TPU) was hot-pressed over the film to improve flexibility. To examine and characterize the nanoparticles and composite films, we used X-ray diffraction (XRD), Fourier Transform Infrared Spectroscopy (FTIR), and scanning electron microscopy with energy-dispersive spectroscopy (EDS). The results verified that the films and nanoparticles were well formed. For a deeper characterization, UV-visible spectroscopy and EMI shielding experiments were conducted for the composite films. The composite films exhibited excellent UV-blocking performance (99.9%) and a total shielding effectiveness (SET) of 13.78 dB in the Ku-band (12–18 GHz) for a thickness of 1 mm. The reflection and absorption mechanisms yield shielding performance through the synergy between conducting (Ni, SWCNT) and magnetic (Fe_3_O_4_) components. These results reveal that the TPU-coated composite film is a promising candidate for multifunctional UV and electromagnetic shielding.

## 1. Introduction

The rapid advances in electronics and communication engineering, coupled with a growing trend toward miniaturization, have enabled the development of highly complex large-scale integrated systems. These systems, known for their complicated circuitry and compact designs, have transformed industries ranging from telecommunications to consumer electronics, but a technical advance always comes with new challenges. Another significant issue is electromagnetic interference (EMI), where electromagnetic waves interfere with the normal functioning of these advanced systems and lead to malfunctions that impact performance and reliability. Apart from operational problems, EMI poses significant health hazards: Prolonged exposure to certain levels of electromagnetic radiation can lead to harmful biological effects. As technological advancement continues to outpace safety measures in unregulated environments, it is crucial to address these emerging issues through further research and new solutions for electronic systems [1,2,3,4].

Due to the necessity of shielding electronic devices from EMI, enhancing radiation immunity, and ensuring functionality within different environments, there has been a growing demand for electrically conductive materials. Metals are the best allies to solve EMI challenges because they reflect electromagnetic waves due to their properties; however, it should be pointed out that both metals and metal-coated materials might reflect off electromagnetic energy due to the poor absorption capacity of such types. The use of metallic materials in modern applications is limited due to disadvantages such as susceptibility to corrosion and high density. On the other hand, Conductive Polymer Composites (CPCs) containing carbon nanotubes as conductive fillers provide a viable alternative to deliver efficient EMI shielding. This class of advanced composites is lightweight, easy to process, and exhibits excellent corrosion resistance, enough to prolong the service life of the parts under diverse environmental conditions. These qualities make them the perfect fit to address current electronic applications where performance, weight, and durability are essential [5,6,7,8,9].

Shielding effectiveness (SE) is described as the absorption or reflection of incoming radiation by shielding materials. This is especially relevant in industries where EMI or ionizing radiation shielding is paramount. Carbon nanotubes (CNTs) have become enormously popular due to their outstanding properties, such as low weight and high electrical conductivity, which can still account for the non-magnetic property of CNTs, making them exploited in many nanotechnology fields. However, the shielding effectiveness of these materials can be enhanced. The introduction of magnetic nanoparticles within CNT-based nanocomposites, along with the proper manipulation of their ratios, provides a promising approach to enhancing the total shielding efficiency. These nanoparticles have magnetic properties that resonate with radiation, both helping to absorb it as well as reflect it back, increasing their efficacy as shielding materials. It exploits the unique properties of CNTs alone or nanocomposites formed with magnetic nanoparticles, leading to a very effective composite material for various shielding applications [10,11,12,13].

In past decades, nickel nanoparticles have been of significant interest to the scientific research community because of their wonderful ferromagnetic behavior and good catalytic performance. Such nanoparticles possess attractive magnetic properties, increasing their efficiency through several applications and spawning many novel disciplines. Another material with potential that can be used to enhance EMI and SE values is magnetite nanoparticles. When it comes to magnetic properties, these particles naturally have strong magnetic attenuation of electromagnetic waves. The ability to absorb and dissipate electromagnetic energy makes them especially useful in applications that require EMI reduction, including electronic devices and communication systems. Their use would result in significant breakthroughs both in technology and in materials science [14,15,16].

These unique magnetic and conductive properties are due to the low presence of chemical treatments needed as surfactants during nanoparticle synthesis, which allows for enhanced electron transfer. Furthermore, we perform the functionalization of single-walled carbon nanotubes (SWCNTs) to tailor their surface properties, enabling better interactions with different materials. Nickel and magnetite nanoparticles are synthesized to allow for the tethering of functionalized SWCNTs, which are systematically combined/embedded in a polyvinylidene fluoride (PVDF) matrix. This method creates a nano-composite film that improves the strength and performance of the materials used. Then, a thermoplastic polyurethane (TPU) coating is employed to enhance the durability and protective properties of this nanofilm. This gives additional protection to the underlying composite and allows the application of sophisticated analytical techniques. In our experiments, the transmittance of UV-blocking and EMI-blocking performance of the nanocomposite film is evaluated.

While both UV shielding and EMI shielding might seem similar in that both relate to the attenuation of electromagnetic radiation, they have fundamentally different technologies operating at different frequency ranges. UV shielding requires interaction with high-frequency electromagnetic radiation in the ultraviolet region (~10^15^ Hz), where attenuation is mostly determined by electronic transitions and the strong absorption of nanofillers like Ni, Fe_3_O_4_, and SWCNTs [16]. Compared with it, EMI shielding is focused on the lower frequency region (GHz), where the conductivity and magnetic loss, as well as interfacial polarization, will dominate the EMI attenuation of the composite [1,2]. Electromagnetic attenuation of different composites mixed with conductive fillers such as SWCNTs and Ni nanoparticles was reported to help charge transport processes, leading to reflection loss enhancement, while hysteresis Fe_3_O_4_ nanoparticles contribute magnetic loss and electromagnetic energy dissipation [4,5]. While the regulation mechanisms are different, their synergistic effects imply that these three nanofillers can work together to achieve not only excellent UV blocking but also the strong EMI shielding performance of the composite film, so that these versatile materials can be used in the electromagnetic protection area.

This integrative and full-cycle strategy is rooted in the theoretical design of advanced materials with targeted functional properties, followed by synthesis through wet-chemical methods, and ultimately characterization.

## 2. Materials

CNTP with OD 1–2 nm, average length 3–8 µm, and high purity (>98 wt%) is available from Nanoshel. Materials used include iron (III) chloride hexahydrate (FeCl_3_·6H_2_O), iron (II) chloride tetrahydrate (FeCl_2_·4H_2_O), ammonium hydroxide (NH_4_OH), hydrazine hydrate (N_2_H_4_·H_2_O), sodium borohydride (NaHB_4_), nickel chloride hexahydrate (NiCl_2_·6H_2_O), hydrazine hydrate (N_2_H_4_·H_2_O), sodium borohydride (NaBH_4_), tetrahydrofuran, polyvinylidene difluoride (PVDF) homopolymer powder from S D Fine Chemicals with 99% purity, India. 3-Amino Propyl Trimethoxy Silane (APTS) from Sigma Aldrich, India. Sulfuric acid (H_2_SO_4_), deionized water, N-Methyl-2-pyrrolidone (NMP), thermoplastic polyurethane(TPU) pellet from Elastollan 1185a, BASF, India.

### 2.1. Nickel Nanoparticles (NNP) Synthesis

The next procedure describes the preparation of nanoparticles of nickel using a liquid-phase chemical reduction process. We dissolved 0.5 g of 99.3 percent pure chemical NiCl_2_·6H_2_O in 20 mL of ethylene glycol first. This solution was stirred at room temperature (600 rpm) using a magnetic stirrer for 2 h, until homogenized and the pH reached 4.2. Next, drop by drop N_2_H_4_·H_2_O, acting as a reducing and complexing agent that controls nucleation and particle growth, was added with vigorous stirring at 80 °C until pH = 12 was achieved. The color of the solution changed gradually from green to light blue, then to light blue-violet, and finally to light pink-violet during this process. After these color changes, the solution was transferred into a round-bottom flask and added to a silicone oil bath at 80 °C; separately, 0.5 g sodium borohydride (NaBH4) was dissolved in 10 mL DI water for colloidal solution preparation. This strong reducing agent facilitated rapid reduction, resulting in a smaller particle size and improved dispersion. Using a burette, the colloidal solution was added slowly to the main mixture. The solution became a metallic black, indicating the production of nanoparticles of nickel suspended in it. Afterward, the black nickel nanoparticles were collected and washed four times with DI water and ethanol. The nanoparticles were dried in a vacuum oven at 70 °C for one hour, then let cool for two hours. The resulting nickel nanoparticles were designated as NNP.

### 2.2. Fe_3_O_4_ Nanoparticles (FNP) Preparation

Iron (III) chloride hexahydrate (FeCl_3_·6H_2_O) and iron (II) chloride tetrahydrate (FeCl_2_·4H_2_O) were dissolved separately in deionized water. These solutions were mixed in a 1:2 molar ratio (using 1 g of FeCl_3_ for every 2 g of FeCl_2_). While stirring, ammonium hydroxide (NH_4_OH) was added dropwise to achieve an approximate pH of 11, and stirred for another 30 min to mix well. The suspension was then centrifuged at 3000–4000 rpm to harvest Fe_3_O_4_ nanoparticles. The washing step was then repeated with deionized water (to remove any residual reagents), followed by rinsing multiple times with ethanol. Finally, the nanoparticles were dried under vacuum at 80 °C to get a fine Fe_3_O_4_ product.

#### 2.2.1. Surface Modification of Fe_3_O_4_ Nanoparticles

The prepared Fe_3_O_4_ nanoparticle was dispersed in 200 mL of H_2_O_2_ solution under bath sonication for 20 min. After bath sonication, the suspended solution was refluxed in a hot oil bath at 106 °C for 4 h. Further, the refluxed solution was washed with de-ionized water in sequence several times to remove the residual reactants. The H_2_O_2_-treated Fe_3_O_4_ was dried in an oven at 80 °C for 24 h and stored in desiccators. Finally, the H_2_O_2_ treated Fe_3_O_4_ nanoparticles were refluxed by APTS at 90 °C for 4 h to achieve –NH_2_ surface functionalization. The resulting solution was then washed with toluene three times to remove the excess amount of APTS yield and dried under vacuum. The functionalized Fe_3_O_4_ nanoparticles are referred to as S-FNP.

#### 2.2.2. Surface Modification of SWCNTs

A uniform dispersion of single-walled carbon nanotubes (SWCNTs) was prepared in an acidic medium. SWCNTs were mixed with concentrated sulfuric acid (H_2_SO_4_) in a reaction vessel and were sonicated for 1 h, with 5min breaks after each 10min sonication. To enhance the oxidation of SWCNTs with H_2_SO_4_, the mixture was refluxed at 200 °C. After acid treatment, the SWCNTs were rinsed thoroughly with deionized water to remove impurities and residual acid. This step was crucial for eliminating harmful acid residues from the modified SWCNTs. The purified SWCNTs were dried at 60 °C in an oven to remove excess water or solvents. The surface-modified SWCNTs are referred to as functionalized single-walled carbon nanotubes (F-SWCNTs).

### 2.3. Preparation of Nano Composite Films

A composite solution was prepared by dispersing 20 wt% of each S-FNP, F-SWCNTs, NNP, and PVDF (40 wt%) into an auxiliary solvent such as N-methyl-2-pyrrolidone (NMP). This mixture was homogeneously stirred to achieve uniform dispersion using ultrasonic irradiation at 20 kHz for 1 h, with an intermittent cycle of 5 min intervals to prevent overheating. When all elements were mixed well, the solution was poured, at room temperature, into a glass Petri dish (100 × 15 mm) for initial curing. The sample was then transferred to a hot-air oven after 2 h and heated for 8 h at 60 °C to ensure complete drying, solvent evaporation, and film formation. The thickness of the obtained nanocomposite film was measured using a screw gauge at various locations across the film and found to be around 2.2 mm.

Next, the sample was gradually cooled from this heat treatment temperature to the room temperature, forming a composite film that will be subsequently referred to as FSNP. TPU pellets (10 wt%) were then dissolved in 8 mL dimethylformamide (DMF) and 2 mL tetrahydrofuran (THF). This solution was stirred for 2 h until completely dissolved using a magnetic stirrer. Subsequently, the TPU solution was applied to the surface of the FSNP film to enhance its flexibility and mechanical properties. Finally, TPU was laminated onto the FSNP film using a lab-scale compression molding machine to obtain a composite film with a thickness of approximately 1 mm, which was used for further characterization and testing.

### 2.4. Characterization

Analyses of Powder X-ray diffraction (XRD) were performed using a Rigaku Rotalflex (RU-200B) instrument, Germany, with Cu Kα radiation (λ = 0.15405 nm) and a nickel filter. The instrument was run at a tube voltage of 40 kV and a tube current of 100 mA. Collecting 2θ angular data from 10° to 80°, the scan rate was set at 5°/min, with a resolution of slow-scan mode: 0.02°. Fourier transform infrared (FT-IR) spectra with wavenumbers 4000 and 400 cm^−1^ were recorded as a KBr disk (JASCO FTIR 6100 spectrometer, Germany). UV-transmission of the composite film in the 280–400 nm range was conducted by a UV-3600 spectrophotometer, following AS/NZS 4399:1996 standards. This included transmission measurements in the UV-A (315–410 nm) and UV-B (290–315 nm) spectra, allowing for the calculation of transmitted intensity (IT), transmittance intensity of ultraviolet radiation, and shielding efficiency (SE). The washing cycles for the 2 × 2 cm composite film samples were conducted using ~100 mL of deionized water. In all, 10 cycles of 15 min each were performed. During this process, a magnetic stirrer (REMI, India) was used for continuous agitation at room temperature. Approximately 1 mL of a commercially available detergent (RIN) was added in each cycle.

## 3. Result and Discussion

### 3.1. Structural, Vibrational, and Morphological Analysis of Nanoparticles

**Figure 1:**
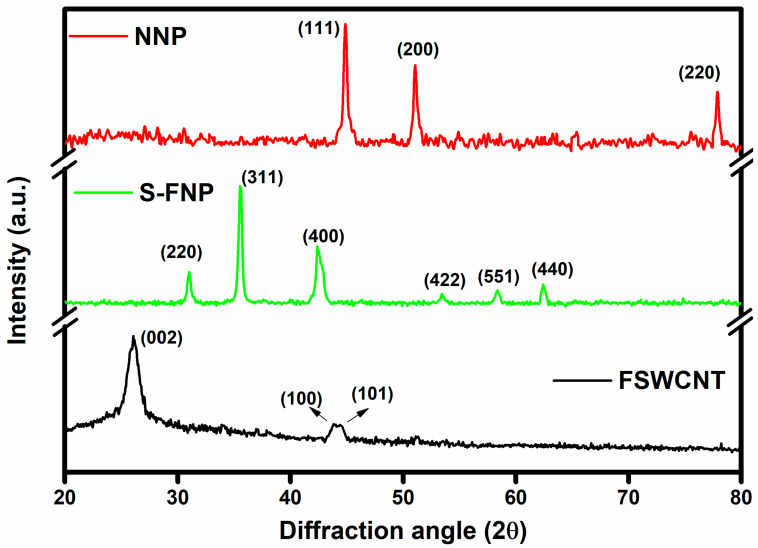
XRD analysis of NNP, S-FNP, and FSWCNT particles.

In Figure 1, it shows the 2θ values number from 20° to 80° for the analysis of X-ray diffraction (XRD) patterns of synthesized nickel nanoparticles (NNP), surface modified magnetite nanoparticles (S-FNP), and SWCNT. The XRD patterns of these nanoparticles have several broad low intensity peaks corresponding to amorphous composition, respectively, or very small size crystal plane domains (<50 nm). In particular, the peaks match that of face-centered cubic (fcc) nickel. The XRD pattern of nickel nanoparticles illustrated in Figure 1 shows three sharp and prominent peaks around 2θ values of 44.6, 52.3, and 78.3 correlated to the (111), (200), and (220) planes, respectively (JCPDS Card No.04-0850) [17]. The presence of ultrafine Ni particles is confirmed by the characteristic peaks associated with fcc nickel crystal structures, with no other oxide or impurity phases. The crystallite size, dislocation density, and microstrain have been obtained from Fourier analysis of each crystalline peak in the XRD patterns using the Debye–Scherrer equation [18], as summarized in Table 1. The main crystallographic phase of the S-FNP sample is defined by the diffraction peaks associated with the (hkl) indices (220), (311), (400), (422), (511), and (440) as per JCPDS Card No. 19-0629. The silane coupling agent exhibited a non-crystalline structure, indicating no diffraction peak. The distinctive peaks for the FSWCNT sample are observed corresponding to (002), (100), and (101) (JCPDS Card No. 41-1487).

**Figure 2:**
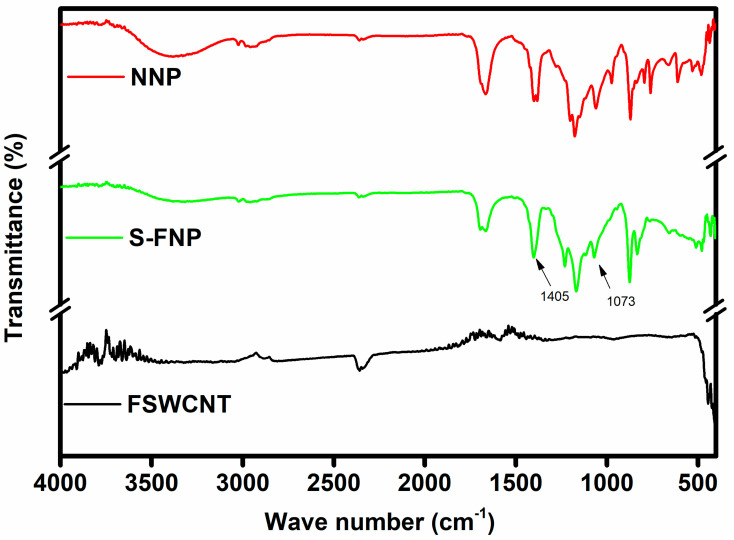
FTIR analysis of NNP, S-FNP, and FSWCNT particles.

The FTIR spectra of NNP, FNP, and FSWCNT synthesized using respective chemical methods (Figure 2), recorded in the range of 4000 to 400 cm^−1^, show more than one significant absorption band. These bands indicate oxide formation and surface-adsorbed species. The Ni nanoparticles synthesized by chemical reduction exhibited a wide band at about 3400–3450 cm^−1^, assigned to the O–H stretching vibration of water or hydroxyl groups on the surface of the nanoparticle in Figure 2; meanwhile, a bending vibration was detected at approximately 1626 cm^−1^ and ascribed to H–O–H bending or absorbed humidity on the nanoparticles. The peaks were at 3212.72, 1623.67, 1402.27, 1076.94, and 463 cm^−1^, which can be assigned to the O-H stretch in Ni (OH)_2_ [19]. The attachment referred to the C-C cross-linking formation (CHC), CH bending, CO stretching, and NiO stretching in CHC. [19,20] Peaks at 1380–1400 cm^−1^ are commonly associated with C–H bending or carbonate species, perhaps attributable to residual or capping organic molecules. In the low wavenumber area, intense absorption bands at approximately 400–500 cm^−1^ (e.g., 445 cm^−1^) relate to Ni–O stretching modes; partial oxidation is noticed, verifying that metal oxide lattices are generated.

The FTIR spectrum usually reveals the sharp absorbance bands from 550 cm^−1^ to 600 cm^−1^ due to Fe–O stretching vibration at both tetrahedral and octahedral, indicating the magnetite formation via spinel structure [21]. A broad band at 3400–3450 cm^−1^ is attributed to O–H stretching vibrations, indicating that adsorbed water molecules or surface hydroxyl groups are present [22]. Weak absorption near 1620 cm^−1^ is due to the bending vibration of H–O–H in surface-adsorbed water. In the presence of surfactants or stabilizing agents, however, other peaks between 1400 cm^−1^ and 1500 cm^−1^ appear due to either C–H units or carbonyl (C=O) functional groups [23], corroborating that organic species are capping the Fe_3_O_4_ nanoparticles. The absorption peaks at 1405 cm^−1^correspond to the C-H stretching and in-plane bending vibrations of -OCH_3_groups within the chains of the silane coupling agent. The characteristics of the Si-O-Si stretching vibration absorption peak are observed within the 1029–1101 cm^−1^ region, with a prominent peak at 1042 cm^−1^in this figure, which confirms that dehydration polymerization has occurred between silane coupling agent molecules. These characteristic peaks, in general, confirm the successful synthesis of Fe_3_O_4_ nanoparticles with a spinel crystal structure and functionalized face.

FTIR spectra analysis shows several presences of absorption bands consistent with oxidation products, and evidence confirming the incorporation of various oxygen-bearing functional groups on the nanotube surface. A wide absorption band centered at 3400–3430 cm^−1^ is a result of the O–H stretching vibration for hydroxyl groups and carboxylic acids on the surface, which proves the existence of surface coated –COOH groups, as well as adsorbed moisture [24]. The weak band around 1710–1730 cm^−1^ is assigned to the C=O stretching vibration of the carboxylic acid group, confirming the direct evidence for the oxidative functionalization of carbon nanotube walls [25]. A weak band near 1620 cm^−1^ is due to the C=C stretching of the graphitic framework and/or H–O–H bending of adsorbed water molecules. Absorptions that appear at 1200–1250 cm^−1^ are attributed to C–O stretching vibrations of the carboxyl or carbonyl groups [26]. Such oxygen-containing functionalization on the SWCNT surface can greatly enhance their chemical reactivity, dispersion, and compatibility with polymer matrices. Hence, FTIR spectroscopy is able to confirm that the carboxylation of SWCNTs was successful and provide evidence for structural changes resulting from the treatment in acid.

**Figure 3:**
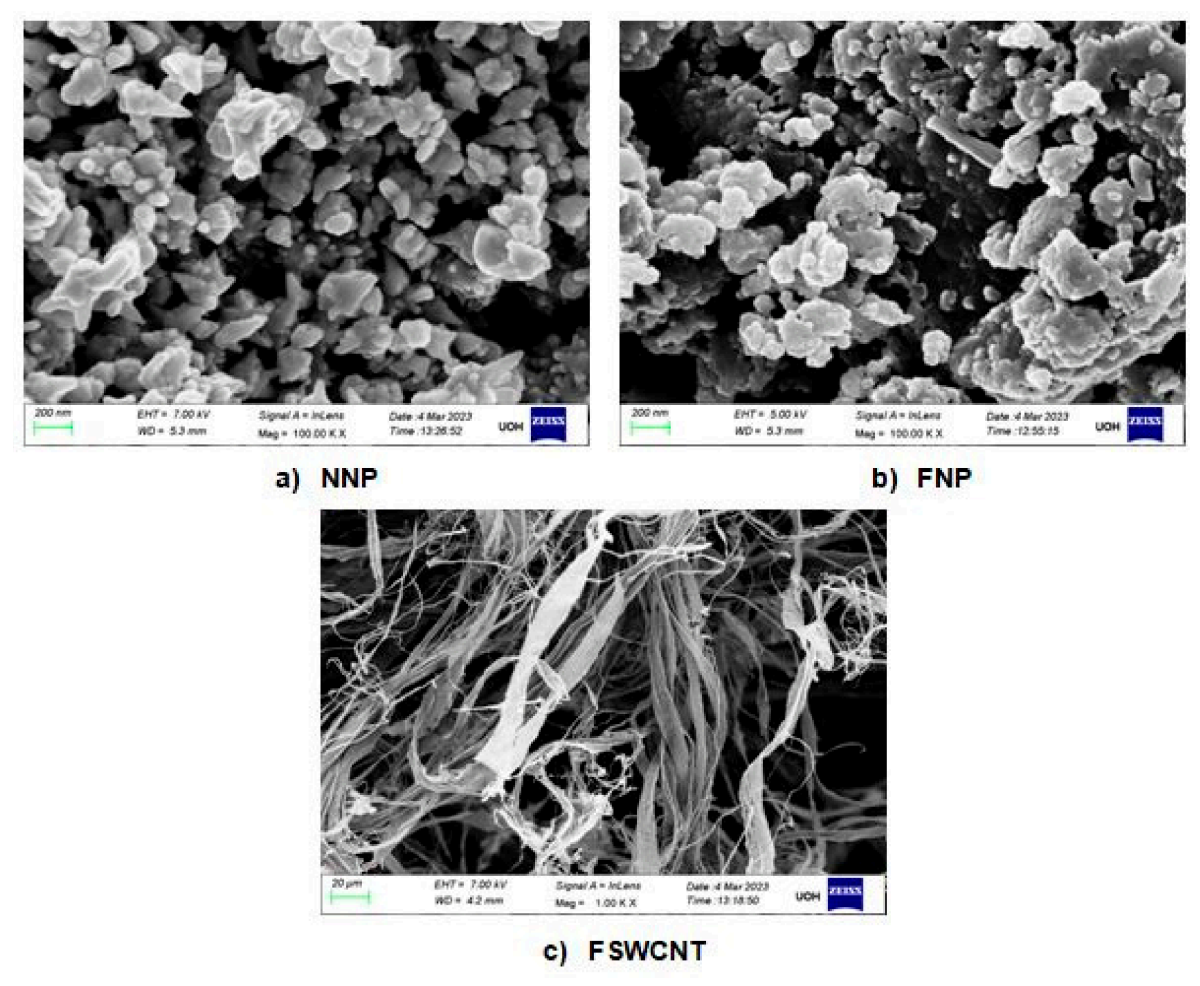
SEM image of (**a**) NNP, (**b**) FNP, and (**c**) FSWCNT particles.

Figure 3 presents representative micrographs captured with a Field Emission Scanning Electron Microscope (SEM), specifically the Zeiss Ultra 55 GEMINI, Germany, showcasing the different particles used in this study. In Figure 3a, the nanoscale network particle (NNP) is shown, measuring about 200 nm. Figure 3b displays the SEM micrograph of the as-received functionalized nanoparticle (FNP), with sizes ranging from 100 to 200 nm. This image indicates that most particles are between 100 and 150 nm and tend to form agglomerates of both NNPs and FNPs. Finally, Figure 3c shows an SEM micrograph of functionalized single-walled carbon nanotubes (FSWCNTs), with an average diameter of approximately 9.5 nm. Additionally, the high-magnification SEM micrograph clearly reveals the number of sidewalls.

**Figure 4:**
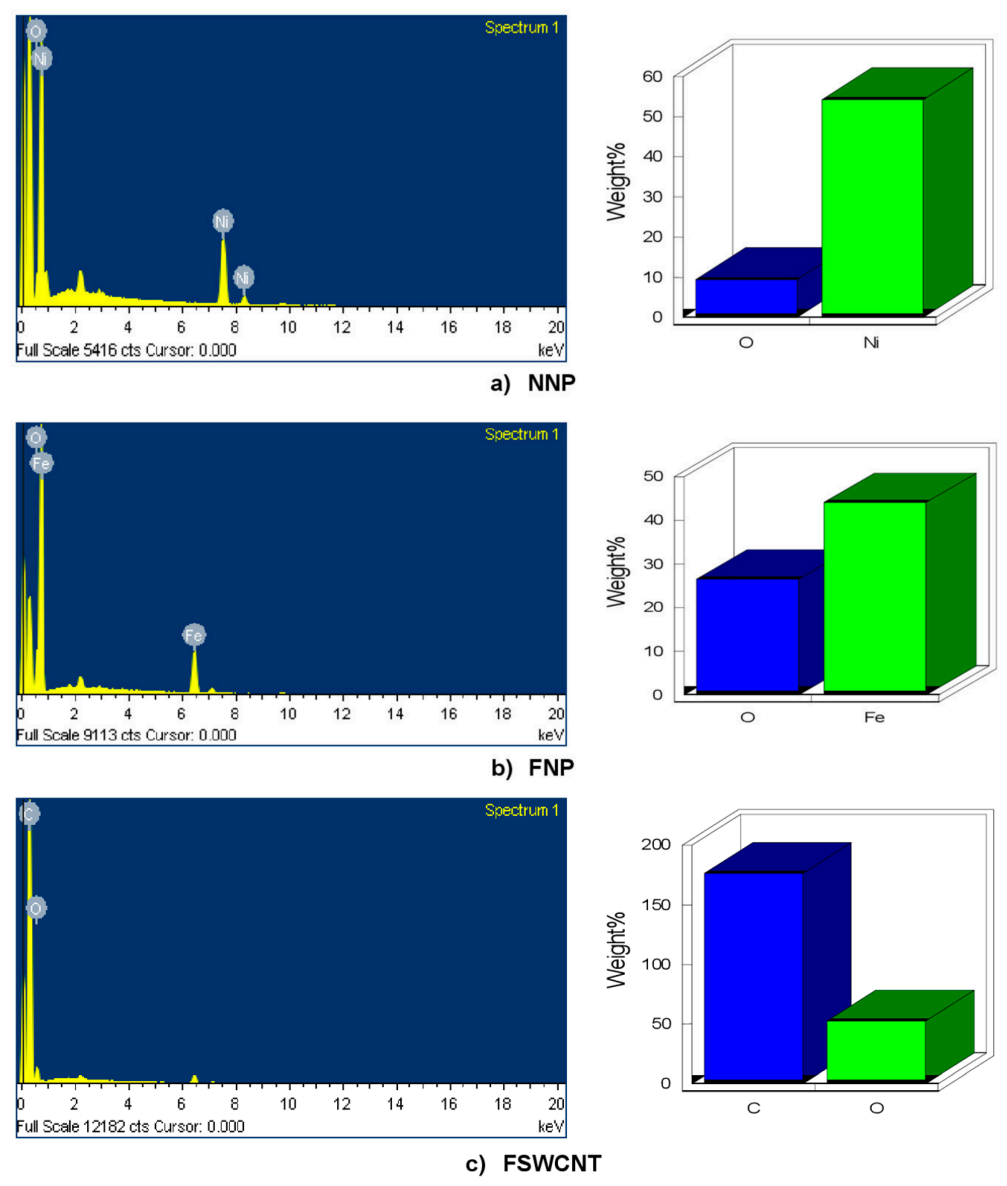
EDS spectra of (**a**) NNP, (**b**) FNP, and (**c**) FSWCNT particles.

The Energy-Dispersive X-ray Spectroscopy (EDS) spectrum, provided by Oxford Instruments, shows the presence of oxygen along with NNP (Figure 4a), FNP (Figure 4b), and FSWCNT (Figure 4c). This indicates that some particles may have undergone partial oxidation, likely during centrifugation and drying. Additionally, the average particle size from SEM analysis matches the crystalline size from XRD analysis, which is significantly smaller than the measured particle size.

### 3.2. Surface Morphology and Elemental Analysis of the Composite Film

**Figure 5:**
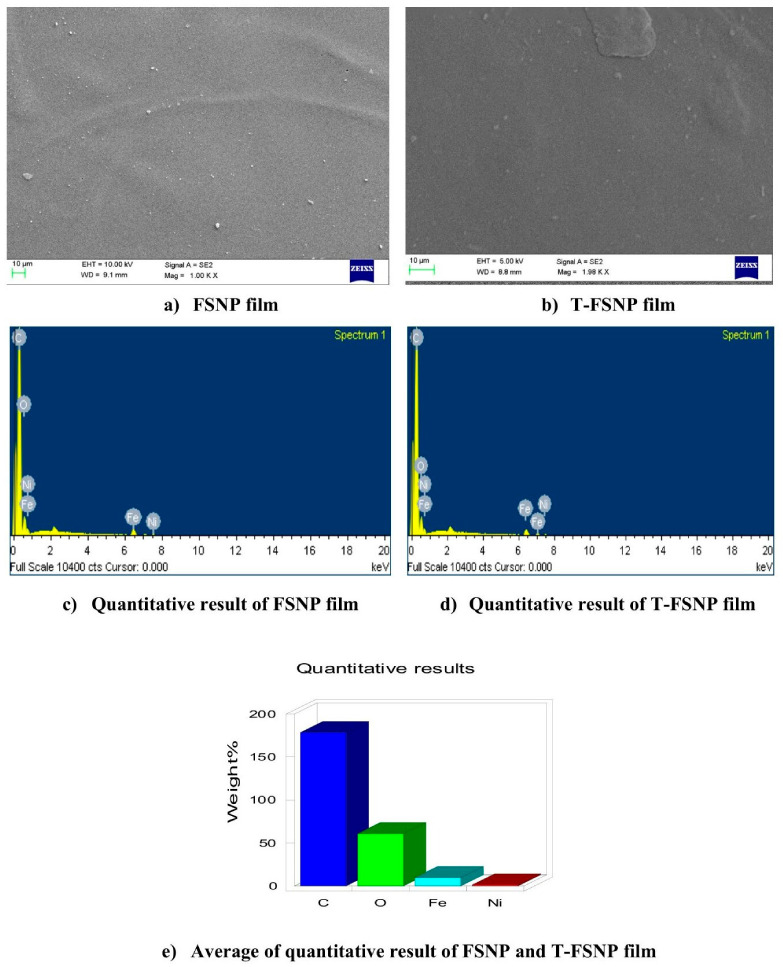
SEM image and EDS spectra of Composite film: (**a**) FSNP film, (**b**) T-FSNP, (**c**) Quantitative result of FSNP film, (**d**) Quantitative result of T-FSNP film and (**e**) Average of Quantitative result of FSNP and T-FSNP film.

Figure 5 presents SEM micrographs of the FSNP and T-FSNP composite films. The SEM images illustrate the surface morphology of the FSNP (Figure 5a) and T-FSNP (Figure 5b). Surface analysis indicates that the FSNP film has a uniform, dense surface. In contrast, the T-FSNP film demonstrates the effective dispersion of nanoparticles throughout the FSNP film and TPU polymer. There are no large agglomerates or cracks seen, suggesting excellent interfacial bonding between Ni, Fe_3_O_4_, and SWCNT fillers and the polymer phase. The smooth surface with few embedded particles further indicates the good dispersion of nanofillers and a continuous conductive and magnetic network across the film. Such morphology constancy determines its mechanical strength, dielectric property stability, as well as shielding effectiveness for UV and EMI.

The elemental compositions of the composite films FSNP (Figure 5c) and T-FSNP (Figure 5d) were confirmed by the EDS spectrum. The Ni and Fe peaks demonstrate the successful embedding of magnetic nanoparticles into the composite film. Strong peaks for the carbon and fluorine elements (Figure 5e) further confirm the supporting compositions of the polymer matrix, CNTs (PVDF/TPU and SWCNT). The observation of the oxygen signal implies the oxide layers’ or hydroxyl groups’ formation on the surface, which correlates with FTIR and XRD information. In general, the elemental distribution provides direct evidence of the hybrid magnetic–conductive composite formation, which immensely benefits efficient EMI shielding through reflection and absorption mechanisms.

### 3.3. Structural Analysis of the Composite Film

**Figure 6:**
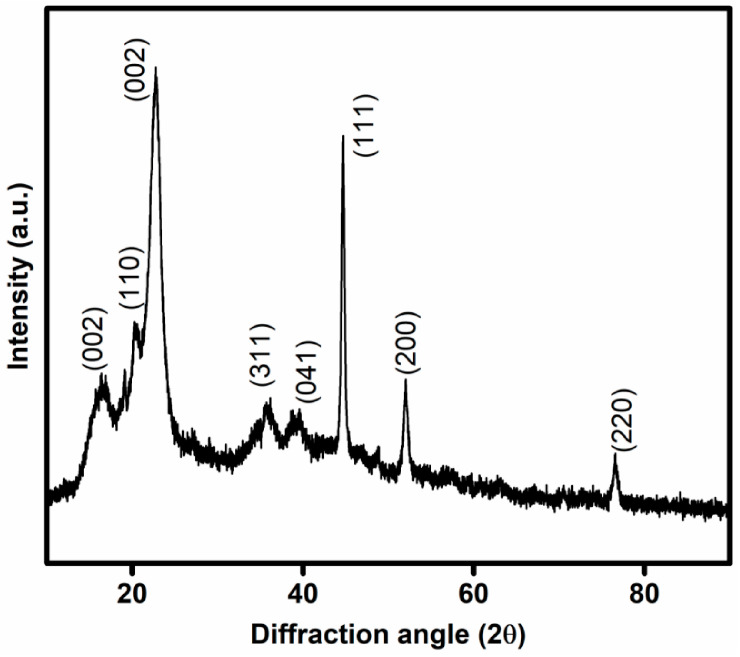
XRD analysis of T-FSNP composite film.

The T-FSNP composite XRD pattern is presented in Figure 6, confirming metallic Ni, magnetite (Fe_3_O_4_), graphitic carbon (SWCNT),and a polymer matrix. The identified peaks are at diffraction angles of 44.5°, 51.8°, and 76° attributed to the planes Ni (111), Ni (200), and Ni (220), which are characteristic of well-crystalline face-centered cubic (fcc) Ni nanoparticles [27]. The features in the ranges of 30–36°, particularly the (311) reflection, are similar to those reported for spinel phase Fe_3_O_4_, confirming the formation of magnetite nanoparticles [28]. A strong (002) reflection at ca 26° indicates the graphitic stacking of SWCNT bundles [29]. Moreover, wide features around 17–20° emphasize the semi-crystalline domains of PVDF embedded in the predominantly amorphous TPU network [30]. The sharper peaks related to Ni, compared to the broader Fe_3_O_4_ peaks, imply that Ni crystallites are larger than the smaller, nanoscale Fe_3_O_4_ particles [31]. The XRD results show that the combination of conductive Ni/SWCNT networks and magnetic Fe_3_O_4_ inclusions aligns with the composite’s mixed reflection and absorption behavior in electromagnetic interference (EMI) shielding.

### 3.4. UV Shielding Property

**Figure 7:**
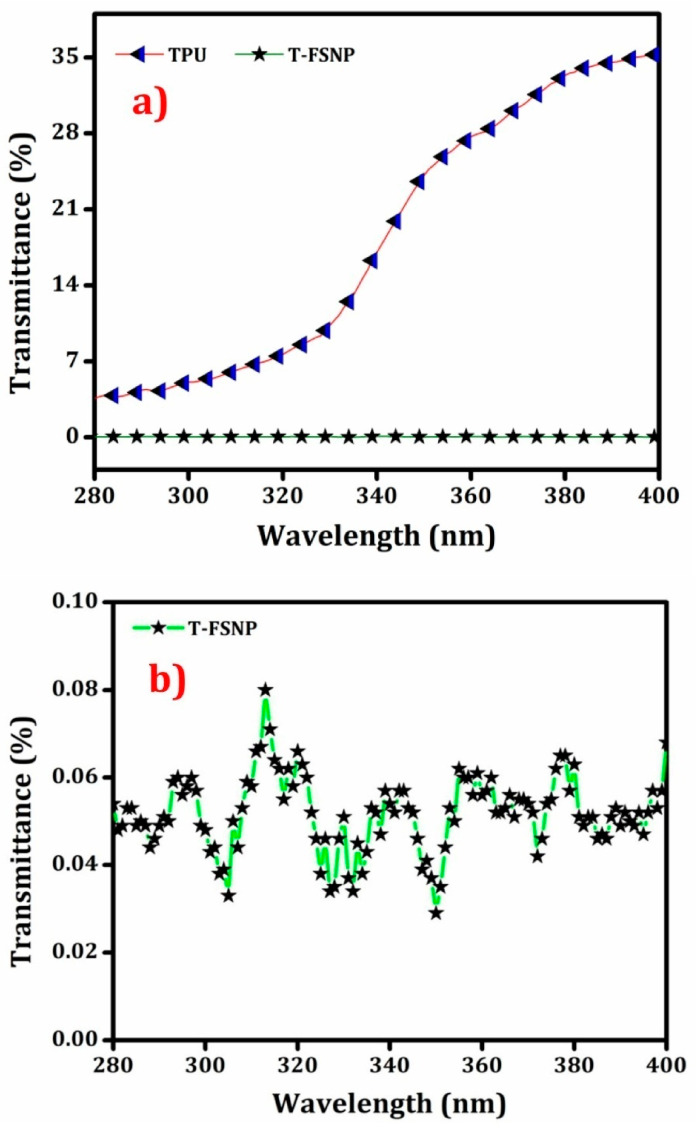
(**a**) UV transmittance spectra of bare TPU film and T-FSNP composite film, (**b**) magnified image of T-FSNP composite film.

The measurements for UV transmittance and blocking were performed with the help of a UV Vis-NIR Spectrophotometer. The UV-blocking analysis of the bare TPU film (used for comparison with the composite film) and the T-FSNP composite film is shown in Figure 5. TPU enhanced with UV-blocking nanomaterials can significantly improve protection against UV radiation. The bare TPU film’s transmittance shows a low region that increases considerably in the near-UV range. Conversely, the composite films exhibit very low transmittance across the UV spectrum, as shown in Figure 7a. The strong UV absorbing characteristics of FSNP may explain this observed phenomenon. The mean of UV-A (315–400 nm) and UV-B (290–315 nm) transmittance data were found to be particularly low in the T-FSNP composite film, which indicates the existence of ultra-violet absorbing materials such as Ni, Fe_3_O_4_, SWCNT, etc. The peaks displayed below 0.1% in the magnified view of the T-FSNP composite film (Figure 7b) imply effective UV absorption by nanomaterials uniformly distributed in the polymer matrix of the composite film.

The UV-blocking percentages of bare TPU film and T-FSNP composite films are listed in Table 2. As shown, the data demonstrates that the T-FSNP composite film offers excellent UV blocking, higher than the bare TPU film. Although both films have a comparably high UV transmittance, the T-FSNP film was found to pass much lower amounts of UV light. Similarly, the T-FSNP average transmission is approximately constant at ∼0.05%. These experimental results demonstrate that T-FSNP is an incredibly strong UV light absorbent compared to bare TPU film and a T-FSNP composite film. Consequently, the TPU-wrapped FSNP film exhibits lower transmission, indicating a stronger shielding effect than the bare TPU film. Based on the transmission spectra, the transmitted intensity (TE) and shielding efficiency (SE) are calculated and listed in Table 3. The table shows that the TE of UV transmission decreases from 4.76% to 0.012%, while the SE of UV increases from 95.24% to 99.988%. This demonstrates that the composite film with FSNP exhibits excellent UV-blocking performance, thanks to the fillers (Ni, SWCNT, and Fe_3_O_4_).

After washing, the UV-blocking percentage and SE of the freestanding films remain similar, indicating the stability of the TPU-wrapped composite films. In this case, T-FSNP composite thin films exhibit lower UV-blocking percentages and SE than those of pre-washed fabrics. The transmittance of the composite film remains constant during the 10th washing cycle due to TPU’s hydrophobic nature. When compared with earlier-washed, coated fabrics, the same blocking percentage was observed. Therefore, the results indicate that the TPU-wrapped FSNP film is an effective shielding material and a cost-efficient option with no potential environmental impact.

### 3.5. EMI Shielding Property

**Figure 8:**
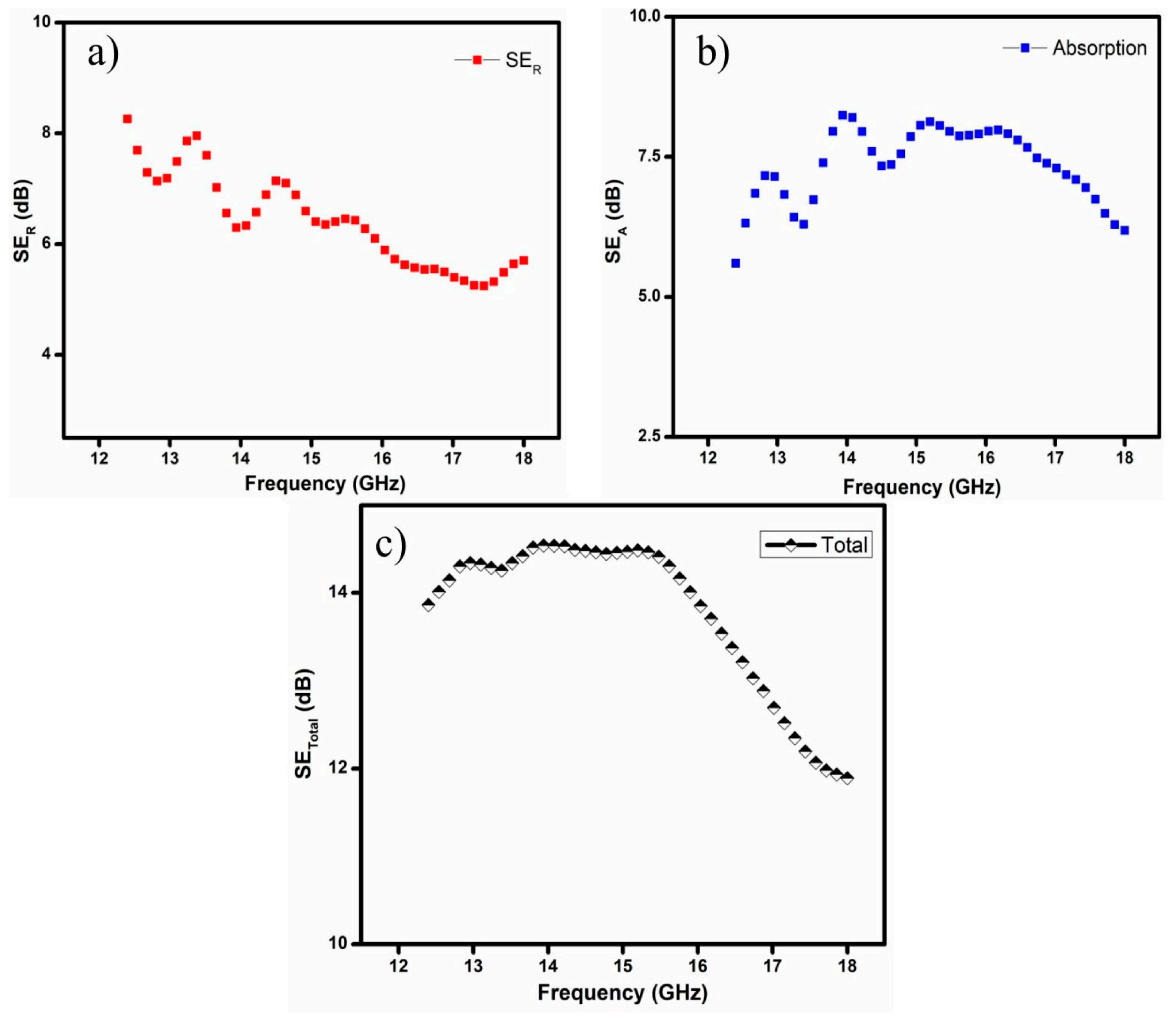
EMI shielding of T-FSNP composite film: (**a**) SE_R_, (**b**) SE_A_, and (**c**) SE_T_.

Electromagnetic shielding involves the usage of conductive materials or materials consisting of electric and magnetic dipoles to minimize electromagnetic waves [32]. Electromagnetic (EM) emission comprises magnetic and electric components that are perpendicular [33,34,35]. Therefore, the shielding material must be both magnetic and conductive. The EMI SE of the T-FSNP composite film was measured in the frequency range of 12 to 18 GHz, using a vector network analyzer from Agilent (Keysight Technologies), model PNA-X (N5242A). The measurements taken on a 1 mm-thick sample are shown in Figure 8a. The electrical conductivity σ = 1/(R_s_t) of the T-FSNP composite film is 8.2 × 10^−3^ S/cm, corresponding to an average shielding effectiveness (SE_T_) of 13.78 dB.TPU was laminated onto the PVDF composite film via compression molding, leading to good interfacial bonding between the layers. At the same time, bare TPU is intrinsically insulating; its presence has a negligible effect on the overall electrical conductivity since charge transport is primarily governed by the interconnected conductive network of NNP, S-FNP, and FSWCNTs embedded within the PVDF matrix. Reflection and absorption are the main aspects that impact the performance of EMI shielding. These aspects are driven by scattering at various surfaces or interfaces, impedance mismatches, and electric dipoles. When Fe_3_O_4_ is in nanoparticle form, the interfacial polarization between the particles and their surfaces is enhanced [36,37,38].

#### Electromagnetic Shielding Mechanism

The electromagnetic interference (EMI) shielding effectiveness of the developed composite is governed by the combined contribution of reflection, absorption, and multiple internal reflections. The total shielding effectiveness (SE_T_) can be expressed as:

(1) $${SE}_{T}={SE}_{R}+{SE}_{A}+{SE}_{M}$$

where SE_R_, SE_A_, and SE_M_ represent shielding due to reflection, absorption, and multiple internal reflections, respectively.

Multiple reflections are insignificant once the absorption loss is greater than 10 dB in real shielding materials. Consequently, the total shielding effectiveness is primarily governed by both reflection and absorption mechanisms [39,40].

The scattering parameters obtained from the vector network analyzer are related to the power coefficients through:

(2) $$R={\left|S_{11}\right|}^{2}$$

(3) $$T={\left|S_{21}\right|}^{2}$$

(4) A=(1−R−T)

Here, the absorption coefficient is denoted as A, the reflection coefficient is denoted as R, and transmission is denoted as T [41]. The shielding components are then calculated using:

(5) $${SE}_{R}=-10\log\left(1-R\right)$$

(6) $${SE}_{R}=-10\log(\frac{T}{1-R})$$

(7) $${SE}_{T}={SE}_{R}+{SE}_{A}$$

Reflection is more or less due to the impedance mismatch between free space and the conductive surface of the material. Using conductive fillers with good coverage and constructing a good interconnectivity state among those fillers will integrate charge carrier mobility while leading to elevated reflection loss [42].

Absorption, on the contrary, is highly dependent on polarization dielectric, interfacial polarization, dipolar relaxation, and conduction loss mechanisms. The formation of heterogeneous interfaces between matrix and conductive fillers contributes to Maxwell–Wagner interfacial polarization, which is beneficial for the attenuation of electromagnetic waves [43]. In addition, the porous and layered microstructure of the composite increases the incident electromagnetic waves propagation path, causing multiple scattering and repeated attenuation in the film surface. These structural features are favorable for energy dissipation as heat and contribute significantly to a stronger absorption loss [44].

To better understand the shielding mechanism of T-FSNP composite film, we calculated the Shielding Effectiveness due to Reflection (SE_R_), Shielding Effectiveness Absorption (SE_A_), and Shielding Effectiveness Total (SE_T_) of TPU/PVDF/Ni/Fe_3_O_4_/SWCNTs nanocomposite for a sample thickness of 1 mm. The results indicate that the SE_R_ was 7.37 dB, the SE_A_ was 6.4 dB, and the SE_T_ value reached 13.78 dB (Figure 8a–c). These findings indicate that the T-FSNP composite effectively reduces EMI, and it is observed that the most crucial mechanism responsible for the EMI shield performance of the T-FSNP composite film is dominated by absorption. Instead of reflecting most incoming electromagnetic waves, the composite mostly absorbs them, significantly boosting its shielding performance. This data is critical to improving the design and use of these types of nanocomposite materials in diverse electromagnetic shielding techniques [45,46].

The evaluation of dominant shielding mechanisms based just on SE_R_ and SE_A_ values has a clear limit since these parameters are not directly equivalent to power coefficients. To be more rigorous in the analysis, scattering parameters (S_11_, S_21_) and electromagnetic properties such as complex permittivity and permeability need to be evaluated, as reported in recent studies [43]. Thus, the EMI shielding behavior of the present composite film has also resulted from the synergistic combination of reflection and absorption mechanisms through conductive and magnetic fillers in the polymer matrix.

## 4. Conclusions

Herein, through a stepwise strategy of chemical reduction, co-precipitation, and surface modification as well as hot-pressing encapsulated with TPU for enhanced flexibility, we present multifunctional Ni/modified (Fe_3_O_4_/SWCNT)/PVDF composite films. The systematic surface modification of modified Fe_3_O_4_ nanoparticles with silane, as well as the functionalization of SWCNTs in sulfuric acid, was necessary for the enhancement of interfacial compatibility as well as uniform dispersion throughout the polymer matrix. XRD, FTIR, SEM, and EDS results confirmed the successful synthesis of the nanoparticles and their effective incorporation into composite films. The developed composite films were prepared and tested using optical and electromagnetic techniques, revealing excellent multifunctional performance with UV-blocking efficiency (99.9%) and EM shielding effectiveness (13.78 dB) in the Ku-band (12–18 GHz). The enhanced UV protection and EMI shielding with the application of magnetic Ni and Fe_3_O_4_ nanoparticles synergistically interacting with conductive SWCNTs embedded in the flexible polymer matrix indicates their overall effectiveness in developing composite films for harnessing next-generation multifunctional devices. The results suggest that the shielding performance arises from the combined effect of reflection and absorption rather than a single dominant mechanism. Thus, flexible composite films have a strong potential for application in advanced fields such as electromagnetic interference shielding and UV shielding.

## Figures and Tables

**Table 1:** Structural Parameters of Nanoparticles.

2θ	FWHM	Crystalline Size (D)	Dislocation Density (δ)	Micro Strain (ɛ)
**NNP**				
44.6	0.8213	11.07	8.18	3.32
52.3	0.3801	24.62	1.65	1.49
78.3	0.1606	67.5	0.22	0.54
**S-FNP**				
31.2	0.106	82.29	0.15	0.45
36.3	0.786	11.25	7.92	3.26
43.9	0.415	21.83	2.09	1.68
54.2	0.024	393.25	0.0064	0.093
58.1	0.055	174.74	0.032	0.21
63.4	0.015	658.34	0.0023	0.056
**FSWCNT (Dominant Peak)**				
26.4	1.452	5.94	28.31	6.17

**Table 2:** UV blocking percentage of bare TPU and T-FSNP composite film, before wash.

S. No.	Sample	T (UV A) (%)	T (UV B) (%)	% Blocking UV A	% Blocking UV B
1	Bare TPU	23.63	05.37	76.37	94.63
2	T-FSNP	0.052	0.054	99.948	99.946

**Table 3:** UV shielding efficiency of TPU and T-FSNP composite film, before washing.

S. No.	Sample	Average Transmittance (%)	Transmitted Intensity	Transmitted Efficiency (TE %)	Shielding Efficiency (SE %)
1	Bare TPU	19.60	0.196	4.76	95.24
2	T-FSNP	0.053	0.00053	0.012	99.988

## Data Availability

The original contributions presented in this study are included in the article. Further inquiries can be directed to the corresponding author.
